# Fall Armyworm in Maize: A Systematic Review of Smallholder Livelihood and Food Security Impacts in Africa

**DOI:** 10.3390/insects17060589

**Published:** 2026-06-04

**Authors:** Constantino Francisco Lhamine, Arsênio Daniel Ndeve, Domingos Raquene Cugala, Pedro Fato, Prince M. Matova, Pedro Silvestre Chauque, Rogerio Marcos Chiulele, Suwilanji Nanyangwe, Mable Chebichii Kipkoech, Kolawole Peter Oladiran, Constantino Tomas Senete

**Affiliations:** 1 Department of Crop Production, Eduardo Mondlane University, Maputo P.O. Box 257, Mozambique; ndevegod@gmail.com (A.D.N.); chiulele.rogerio@gmail.com (R.M.C.); suwilanjinanyangwe02@gmail.com (S.N.); mablekipkoech13@gmail.com (M.C.K.); oladirankolawole5@gmail.com (K.P.O.); 2 Centre of Excellence in Agri-Food Systems and Nutrition (CE-AFSN), Eduardo Mondlane University, Praca 25 de Junho Edificio da Reitoria 5° Andar, Maputo P.O. Box 257, Mozambique; dcugala@gmail.com; 3 Agricultural Research Institute of Mozambique (IIAM), Maputo P.O. Box 3558, Mozambique; fatomagunge@gmail.com (P.F.); chauquepedro@hotmail.co.uk (P.S.C.); senetecosta@gmail.com (C.T.S.); 4 Department of Crop Protection, Eduardo Mondlane University, Maputo P.O. Box 257, Mozambique; 5 Department of Research and Specialist Services, Crop Breeding Institute, 5th Street Extension, Harare 00263, Zimbabwe; 6 International Maize and Wheat Improvement Center (CIMMYT), P.O. Box MP163, Mount Pleasant, Harare 00263, Zimbabwe; 7 Department of Plant Sciences, University of the Free State, Bloemfontein 9300, South Africa

**Keywords:** fall armyworm, *Spodoptera frugiperda*, maize, host-plant resistance, genetic improvement, smallholder agriculture, systematic review, sub-Saharan Africa

## Abstract

Fall armyworm, *Spodoptera frugiperda* (J.E. Smith), has emerged as one of the most damaging invasive pests affecting maize production and household food security across sub-Saharan Africa since its first detection in 2016. This systematic review synthesizes empirical evidence published between 2016 and 2025 to assess the agronomic, livelihood, and food security impacts of FAW on smallholder farming systems across Eastern, Southern, Western, and Central Africa. Following the Preferred Reporting Items for Systematic Reviews and Meta-Analyses (PRISMA) guidelines and the Population, Intervention, Comparison, Outcome, Time, and Setting (PICOTS) framework, 20 studies (17 empirical and 3 contextual) were identified through comprehensive searches of academic databases and institutional repositories and were included in the final synthesis after methodological screening. The evidence indicates that FAW invasion causes substantial maize yield losses ranging from approximately 20% to 50%, with the greatest reductions reported in rain-fed systems with limited access to pest management technologies. Infestation rates frequently exceeded 50%, particularly during early invasion phases. Beyond agronomic losses, several studies reported reduced household income, constrained food availability, and livelihood disruptions, including increased labor requirements, higher production costs, and reliance on short-term coping strategies. Only a small proportion of studies (*n* = 4) directly assessed nutrition-related indicators, but the available evidence indicates declines in dietary diversity in severely affected communities. Overall, the agronomic impacts of FAW are consistently documented across regions, whereas the socioeconomic and nutrition outcomes remain comparatively underreported, indicating a significant evidence gap. These findings highlight FAW as both an agronomic and livelihood challenge, underscoring the need for integrated pest management strategies, strengthened extension services, and coordinated policy responses to safeguard food and income security among smallholder farmers in Africa.

## Simple Summary

Fall armyworm, *Spodoptera frugiperda* (J.E. Smith), has become one of the most destructive pests affecting maize production in Africa since its first detection in 2016. Smallholder farmers are particularly vulnerable because they often have limited access to effective pest management options. This systematic review synthesizes evidence from published studies to understand the agronomic, livelihood, and food security impacts of fall armyworm in African maize systems. The findings show that the pest causes substantial yield losses and contributes to reduced household income and food availability in many regions. However, the socioeconomic and nutrition-related impacts remain insufficiently documented. These results highlight the need for integrated pest management strategies and the development of maize varieties with improved resistance to support smallholder farmers across Africa.

## 1. Introduction

The fall armyworm, *Spodoptera frugiperda* (J.E. Smith), is a highly polyphagous lepidopteran pest native to tropical and subtropical regions of the Americas. It was first detected in West Africa in early 2016, marking the beginning of one of the most significant biological invasions in recent agricultural history [1]. Within two years, FAW had spread across more than 40 African countries, facilitated by favorable climatic conditions, wind dispersal, and the pest’s exceptional reproductive capacity [2]. Subsequent invasions into Asia (2018) and Oceania (2020) confirmed its global pest status, posing a persistent threat to food production systems worldwide [3,4].

FAW attacks over 350 plant species, including rice, sorghum, millet, and sugarcane, but its economic impact is most severe in maize, the primary staple crop for millions of smallholder farmers across sub-Saharan Africa (SSA) [5,6]. The pest’s rapid spread and high feeding capacity have caused substantial yield losses, empirical studies estimate reductions ranging from 20% to over 50%, depending on infestation intensity, agroecological conditions, and management responses [7,8,9]. These losses translate into significant economic costs. For instance, the Centre for Agriculture and Bioscience International [2] estimated annual maize yield losses across Africa at between USD 2.5 and 6.2 billion, threatening the food and income security of over 200 million people who depend on maize as a dietary staple and cash crop.

Beyond direct agronomic losses, FAW has broader socioeconomic and food security implications. Smallholder farmers, who constitute more than 80% of the farming population in SSA, are particularly vulnerable because they rely on rain-fed production systems, limited access to extension services, and inadequate pest management infrastructure [9,10]. In affected communities, FAW outbreaks have led to increased production costs, reduced household income, and greater food insecurity. Some households have resorted to coping strategies such as selling assets, reducing meal frequency, or shifting to less preferred staple foods [2,11]. These dynamics highlight FAW as not merely an agronomic challenge but a multidimensional livelihood and food security crisis.

While numerous country-level studies have been conducted documenting pest incidence, yield loss, and control practices, the evidence remains fragmented and context-specific. Recent comprehensive reviews, such as [12], provide important insights into the biology, ecology, and management of fall armyworm in Africa, including advances in host-plant resistance and integrated pest management strategies. However, these studies primarily emphasize biological and agronomic aspects, with comparatively limited focus on the combined impacts of FAW on smallholder livelihoods and food security outcomes across diverse agroecological contexts. Variations in study design, outcome measurement, and data reporting continue to make it difficult to derive a comprehensive understanding of FAW’s aggregate impact across Africa.

Given the magnitude of the problem and the diversity of existing research, a systematic synthesis of evidence is essential to clarify the extent of FAW’s impact and guide future management strategies. Therefore, the objectives of this review are to: (1) systematically identify, collate, and synthesize published evidence on the impacts of FAW invasion on food security and smallholder livelihoods in Africa; (2) compare findings on food security outcomes across different agroecological zones and farming systems; and (3) identify knowledge gaps and provide recommendations for research, extension, and policy interventions to strengthen resilience among smallholder farmers. The key research questions are: (1) What is the documented impact of fall armyworm invasion on maize yields and food availability in Africa? (2) How has FAW invasion affected yield loss in maize and household livelihoods, including income, labor use, and coping strategies? (3) What contextual factors (e.g., location, farming system, pest management practices) influence the severity of FAW impacts on maize yield and production?

By consolidating quantitative and qualitative findings from 2016 to 2025, this systematic review contributes to a more integrated understanding of FAW’s multidimensional impacts. It provides a baseline for harmonizing future research approaches and informing sustainable pest management policies aimed at protecting both crop productivity and rural livelihoods across sub-Saharan Africa. Synthesizing evidence on agronomic and livelihood impacts of fall armyworm, this review also provides an important foundation for guiding genetic improvement strategies, particularly the development and deployment of maize varieties with host-plant resistance, which represents a sustainable long-term approach to pest management in African smallholder systems.

## 2. Methods

### 2.1. Scope of the Review

This systematic review focuses on empirical and contextual studies assessing the impact of FAW invasion on maize production, household livelihoods, and food security across Africa. The geographical scope includes sub-Saharan African countries where FAW infestations have been reported since its first detection in Nigeria in 2016. Studies were included from Eastern, Southern, Western, and Central Africa, encompassing diverse agroecological zones such as the savanna, humid tropics, and mixed maize-based farming systems. The review period spans 2016–2025, corresponding to the post-invasion decade that captures both the immediate and long-term effects of FAW on African agriculture. Eligible studies comprised empirical and review-based studies, including peer-reviewed journal articles, theses, and institutional reports, providing quantitative or qualitative evidence on FAW impacts at household, community, or regional levels. The use of English-language studies reflects the predominance of English in international scientific publishing. However, we acknowledge that this may limit the representativeness of the evidence base, particularly for Francophone and Lusophone regions of sub-Saharan Africa, and this potential language bias is considered a limitation of the review. The review design followed the Population, Intervention, Comparison, Outcome, Time, and Setting (PICOTS) framework to ensure methodological transparency and consistency. In the context of this review, the intervention/exposure was defined as the occurrence of FAW infestation or the implementation of related management responses, while the comparison included pre-invasion versus post-invasion conditions, affected versus less-affected production areas, or differences in farmer management strategies. This application of PICOTS is consistent with its use in observational evidence synthesis and systematic reviews in agricultural and public health research [13]. The Population consisted of smallholder maize farmers or farming households within sub-Saharan Africa; the Intervention was exposure to FAW infestation or related management practices; Comparisons were made between pre and post-invasion conditions, affected versus unaffected areas, or differing pest management strategies; Outcomes included maize yield loss, infestation rates, household income, food availability, and nutrition indicators; the Time frame covered the years 2016–2025; and the Setting referred to smallholder or subsistence agricultural systems typical of SSA [1,2]. The PICOTS framework used to guide study selection and evidence synthesis is summarized in Table 1. 

All stages of this review adhered to the Preferred Reporting Items for Systematic Reviews and Meta-Analyses (PRISMA) guidelines [13], which provide a standardized framework for documenting identification, screening, eligibility, and inclusion of studies. A detailed PRISMA flow diagram was used to illustrate the literature selection process. Data extraction was conducted using a structured form aligned with the review objectives, and study quality was assessed according to methodological rigor, clarity of outcome measurement, and relevance to food security and livelihood indicators. These steps ensured a systematic and reproducible synthesis of FAW’s agronomic and socioeconomic impacts across Africa.

In addition, the review was designed to generate evidence relevant to breeding and genetic improvement strategies by identifying patterns of pest pressure, yield loss, and vulnerability across agroecological contexts.

### 2.2. Review Protocol and Reporting Standards

This systematic review was undertaken in line with the PRISMA guidelines and followed a protocol registered in the Open Science Framework (OSF) available at: https://doi.org/10.17605/OSF.IO/PTQEK.

### 2.3. Search Strategy

A comprehensive and systematic literature search was conducted to identify peer-reviewed studies and relevant reports examining the impacts of fall armyworm on maize production, food security, and smallholder livelihoods in Africa. The search targeted multiple academic databases, including Google Scholar, ScienceDirect, and SpringerLink, selected for their extensive coverage of agricultural, entomological, and socioeconomic research [2]. To ensure completeness and minimize publication bias, additional grey literature was retrieved from institutional repositories such as the Food and Agriculture Organization (FAO), the Centre for Agriculture and Bioscience International (CABI), and the United States Agency for International Development (USAID)**,** as these agencies have published foundational reports on FAW monitoring and management in Africa [2].

The search strategy was guided by the review’s objectives and structured around combinations of keywords and Boolean operators to capture all relevant studies. The main search strings included the following terms: “fall armyworm” OR “*Spodoptera frugiperda*”; “food security” OR “livelihoods” OR “yield loss”; “maize” OR “corn”; and “Africa” OR specific country names (e.g., Mozambique, Kenya, Zambia, Ghana, and Nigeria).

These search terms were refined iteratively to account for differences in database syntax and to improve the specificity of results [5,11]. Searches were limited to English-language publications and the period 2016–2025, corresponding to the years following FAW’s first confirmed detection in Africa [1] through to the most recent studies.

All retrieved citations were exported to Mendeley Reference Manager for organization, de-duplication, and screening. Reference lists of included studies and review papers were also manually examined to identify additional eligible studies not captured by database searches [14,15]. The screening process followed a two-stage approach: (1) title and abstract screening to exclude clearly irrelevant records, and (2) full-text assessment of remaining studies against predefined eligibility criteria based on the Population–Intervention–Comparison–Outcome–Time–Setting (PICOTS) framework.

This systematic and replicable search strategy ensured broad coverage of empirical and contextual evidence on the biological, agronomic, and socioeconomic impacts of FAW in sub-Saharan Africa, consistent with best practices for evidence synthesis [13].

### 2.4. Identifying Other Useful Sources

The search was restricted to peer-reviewed journal articles, conference papers, thesis and relevant agency reports published between 2016 and 2025 in English. Reference lists of included papers were also screened for additional relevant studies.

### 2.5. Eligibility Criteria

The eligibility criteria applied for study selection are summarized in Table 2, based on the PICOTS framework and the objectives of the review.

### 2.6. Study Design, Publication Criteria, and Scope

To ensure methodological rigor and relevance, the review included sources presenting original empirical data, as well as selected review and synthesis studies used for contextual interpretation. These sources were selected because they provide verifiable evidence derived from systematic research methods, such as field surveys, household assessments, and observational or quasi-experimental studies [5,11]. Institutional and technical reports published by FAO, CABI, and USAID were also included due to their critical role in documenting early FAW outbreaks and their relevance to food security and livelihood outcomes [2,15].

The review incorporated a broad range of empirical research designs to capture the diverse ways in which FAW invasion has been studied across Africa. Eligible studies included quantitative observational designs, such as household surveys, cross-sectional analyses, and longitudinal or panel studies, as well as quasi-experimental studies including before-and-after comparisons and difference-in-differences approaches. Qualitative and mixed-methods studies were also included when they assessed implications for livelihoods, coping strategies, and food security outcomes.

To maintain methodological alignment with the review objectives, studies focusing exclusively on laboratory, greenhouse, or pesticide efficacy trials without measurable livelihood or food security outcomes were excluded. Similarly, editorials, opinion pieces, newsletters, and conference abstracts without full methodological details were omitted.

Only studies published from 2016 onwards, corresponding to the first confirmed detection of FAW in Africa, were considered. In addition, only studies conducted in Africa, with a focus on smallholder or subsistence farming households, were included. Multi-country studies were considered only when African results were clearly disaggregated.

### 2.7. Study Quality Assessment

The quality of all included studies was assessed systematically to ensure reliability and relevance to the objectives of this review. The methodological quality of the included studies was evaluated using the Critical Appraisal Skills Programme (CASP) cohort checklist, which is widely applied for assessing observational and non-randomized research in public health and agricultural systems. The checklist examines key aspects of study validity, including clarity of research objectives, appropriateness of study design, sampling strategy, measurement of exposure and outcomes, consideration of confounding variables, precision of estimates, and applicability of findings to the target population.

Because this review included a mixture of primary empirical studies and secondary evidence (e.g., literature reviews, modelling analyses, and descriptive outbreak reports), only primary empirical studies were subjected to formal risk-of-bias assessment. Secondary sources were retained for contextual interpretation but were not included in the methodological scoring process, consistent with best practice in evidence synthesis.

Each empirical study was independently evaluated against CASP criteria and assigned a cumulative score reflecting the extent to which methodological standards were met. Based on these scores, studies were categorized into three levels of risk of bias: low risk (strong design, objective outcome measurement, and appropriate analytical methods), moderate risk (observational design with some methodological limitations such as reliance on self-reported outcomes or incomplete adjustment for confounders), and high risk (weak measurement strategies, limited methodological transparency, or absence of statistical rigor).

The assessment criteria were adapted from established guidelines for evaluating observational and mixed-methods research in agricultural and public health contexts [16,17]. Each study was examined first for the clarity of its research question or objective, with attention to whether the stated aim was clearly articulated and directly relevant to FAW impacts on maize, food security, or smallholder livelihoods. Clear research aims are essential for assessing the internal validity and coherence of empirical studies, particularly in multidisciplinary fields such as pest management and rural development [13].

The appropriateness of the study design was then evaluated in relation to the stated research objectives. Quantitative studies were assessed based on whether cross-sectional, longitudinal, or quasi-experimental approaches were suitable for addressing questions related to yield loss, infestation severity, or household welfare outcomes [5,18]. Qualitative studies were appraised for methodological rigor in the use of interviews, focus groups, or ethnographic case studies, ensuring that they systematically captured farmer experiences and community-level responses to FAW invasion [9,19].

Assessment of population and sampling procedures focused on whether the target population was clearly described and representative of smallholder maize-growing households. Studies were examined for sufficient detail on sampling frames, selection criteria, and inclusion or exclusion criteria, consistent with recommendations for observational research in developing-country settings [2,20].

The evaluation further considered the definition and measurement of FAW exposure or intervention. This included whether studies clearly defined infestation levels, outbreak years, farmer-reported incidence, or management responses such as pesticide use or integrated pest management practices. Clear exposure assessment is crucial for attributing outcomes to FAW rather than to unrelated agricultural shocks such as drought or socio-economic constraints [6,15].

In reviewing outcome measurement, the assessment examined whether studies used valid and reliable indicators for food security and livelihood outcomes, including maize yield, dietary diversity, income changes, coping strategies, and household food availability. Strong emphasis was placed on outcomes explicitly linked to FAW impacts, consistent with best practices for impact evaluation [13].

The quality of data collection and analysis was also evaluated. This included determining whether data collection methods such as structured household surveys, validated questionnaires, field observations, and interview protocols were appropriate and transparently described. Analytical approaches were examined for their suitability to the data type, including regression modeling for quantitative studies and thematic or content analysis for qualitative studies [5,14].

Lastly, attention was given to bias and confounding, assessing whether studies acknowledged potential sources of bias such as selection bias, recall bias, or reporting bias, and whether confounding factors such as drought, crop diseases, or market shocks were accounted for in the analysis. Transparent reporting of findings, presentation of sufficient detail, and discussion of study limitations were also key components of the quality assessment, consistent with PRISMA 2020 guidance [13].

### 2.8. Data Extraction and Synthesis

Data extraction was conducted systematically using a standardized form developed in alignment with the review objectives and the PICOTS framework. For each eligible study, detailed information was recorded, including bibliographic data (authors, year, title, and journal), the country and agroecological context, and the study design and methodology, such as household surveys, field observations, cross-sectional analyses, or mixed-methods approaches [1,5]. Extracted outcomes included quantitative measures such as maize yield loss (%), FAW infestation rate, household income effects, and food availability, as well as nutrition indicators were reported.

In addition to numerical outcomes, qualitative findings were recorded to capture farmer perceptions, coping strategies, management practices, and broader livelihood impacts. Each study’s key findings were summarized in terms of the magnitude and direction of FAW impacts, facilitating comparison across geographical regions and methods. Study limitations and author-reported recommendations were also documented to inform the interpretation of results and highlight areas requiring further research [2,9].

Extracted data were synthesized narratively, supported by descriptive statistics where appropriate. Given the heterogeneity in study designs, outcome measures, and analytical approaches, formal meta-analysis was not feasible; instead, the synthesis followed an integrative methodology consistent with PRISMA recommendations for reviews combining qualitative and quantitative evidence [13]. Quantitative interpretation and outcome synthesis were therefore based exclusively on empirical studies, while contextual studies were used to support interpretation.

The data extraction process covered 20 eligible studies, including 17 empirical studies and 3 contextual studies published between 2016 and 2025, as summarized in the data extraction table. These studies provided quantitative or descriptive evidence on the impacts of fall armyworm in smallholder maize systems across Africa. Data extraction was conducted independently by two reviewers using predefined criteria to ensure consistency, and any discrepancies were resolved through discussion and consensus.

To assess reporting completeness, the extracted dataset was analyzed to identify which outcomes were explicitly reported versus inferred or not assessed. Among the 20 studies, 75% reported yield loss data and 71% reported infestation rates. Socioeconomic outcomes were less consistently measured, with 50% reporting household income effects and 46% including food availability indicators. Nutrition outcomes were rarely assessed, appearing in only 17% of studies. These patterns indicate a strong empirical emphasis on agronomic impacts of fall armyworm, alongside important gaps in the measurement of livelihood and nutrition outcomes in smallholder farming systems.

## 3. Results

### 3.1. Study Selection

**Figure 1:**
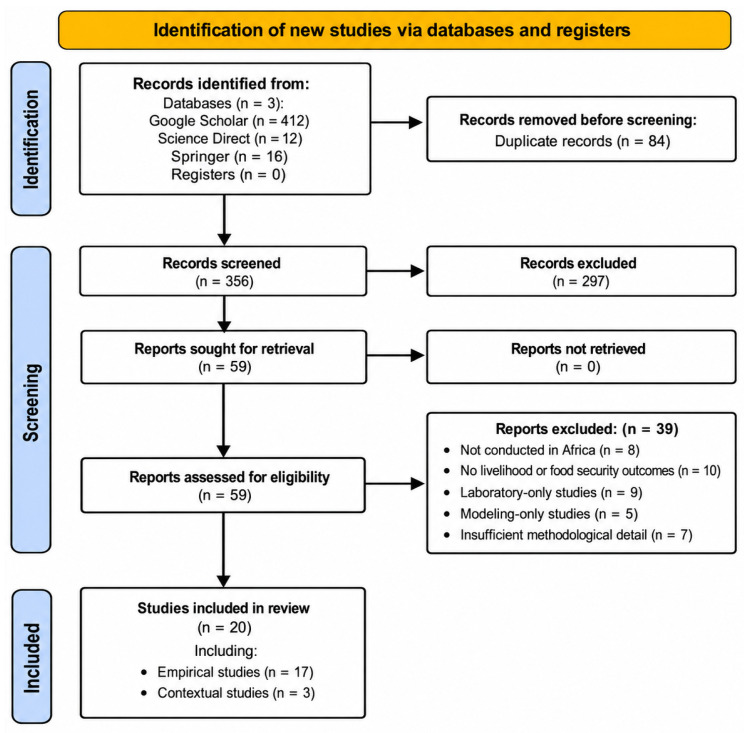
PRISMA flow diagram of the study selection process.

A systematic search of electronic databases and institutional repositories identified a total of 440 records. After removal of 84 duplicates, 356 records remained for title and abstract screening. Of these, 297 were excluded for not meeting the predefined inclusion criteria. Sixty-five full-text articles were assessed for eligibility, and 39 were excluded for reasons including non-African study location, absence of livelihood or food security outcomes, laboratory-only focus, modeling-only studies, or insufficient data. Ultimately, 20 studies met the eligibility criteria and were included in the qualitative synthesis (Figure 1).

### 3.2. Results of the Study Quality Assessment

**Figure 2:**
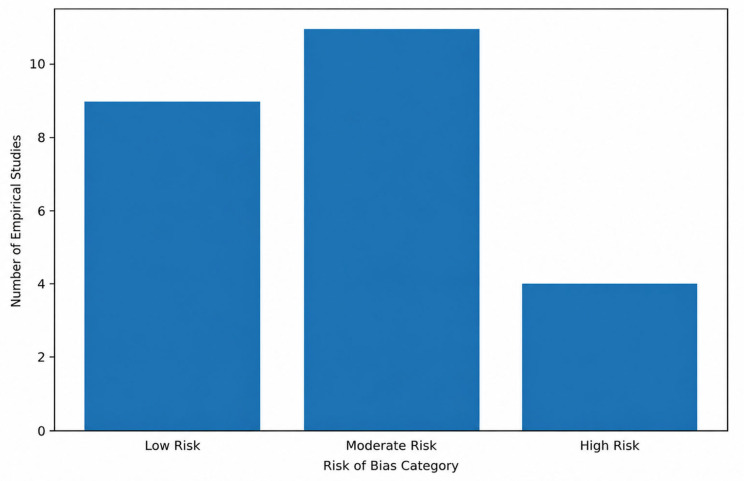
Risk of bias summary of primary empirical studies.

The results of the study-level appraisal are provided in Supplementary Table S1, while a graphical summary of the distribution of risk categories across studies is presented in Figure 2. Overall, most empirical studies exhibited moderate methodological rigor, reflecting the predominance of cross-sectional and survey-based designs in fall armyworm impact research across Africa.

### 3.3. Geographic Distribution of Studies

Table 3 summarizes the included studies by geographic scope, countries covered, and type of research. The dataset comprises 20 studies, including 17 empirical studies (16 country-specific and 1 multi-country study) and 3 contextual studies (review, modeling, and program reports). Mozambique accounts for the highest number of country-specific empirical studies, while several countries are represented by only one study. Zambia and Kenya are also represented by multiple studies, reflecting their importance as major maize-producing countries affected by fall armyworm.

The geographic distribution of empirical studies reveals an uneven concentration of research across Africa. Most countries are represented by a limited number of studies, highlighting substantial gaps in country-level empirical evidence. In addition to country-specific studies, one study adopted a multi-country perspective, covering multiple African countries simultaneously. While such studies provide broader insights, the limited number of country-specific assessments suggests that localized evaluations of fall armyworm impacts on yield, livelihoods, and food security remain insufficient in many parts of Africa.

**Figure 3:**
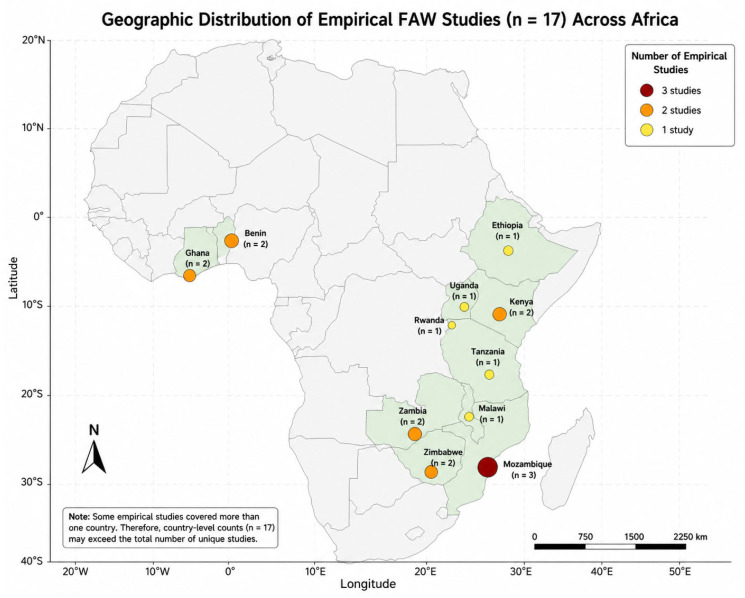
Geographic distribution of empirical FAW studies (*n* = 17) across Africa. Circle size represents the number of empirical studies conducted in each country. Countries shaded in green indicate the locations where empirical studies were conducted. Some studies covered more than one country; therefore, country-level counts may exceed the number of unique studies. Locations are approximate and based on country centroids.

Overall, the 20 included studies consist of 17 empirical studies and 3 contextual studies, as presented in Table 3, while the spatial distribution of empirical studies (*n* = 17) is illustrated in Figure 3.

### 3.4. Geographic Distribution Map

Figure 3 illustrates the geographic distribution of empirical studies (*n* = 17) across sub-Saharan Africa. The spatial pattern reveals a clear concentration of research activity in Eastern and Southern Africa, particularly in countries such as Mozambique, Kenya, and Zambia. In contrast, several regions, including Central and Northern Africa, remain underrepresented in terms of empirical evidence. This uneven distribution highlights important geographic gaps in research coverage, suggesting that the current evidence base may not fully capture the diversity of agroecological and socioeconomic conditions across the continent. The map therefore provides a spatial perspective that complements Table 3, which summarizes the full set of included studies.

### 3.5. Characteristics of Included Studies

Supplementary Table S1 and Figure 2 summarize the main characteristics and findings of the studies included in this review such as FAW exposure metrics, measured outcomes, effect sizes, and management practices reported in each study. It also captures key socioeconomic and food security indicators such as yield loss percentages, infestation rates, income effects, and food availability along with reported limitations. Altogether, these data provide an integrated overview of the evidence base on how FAW invasion has affected maize production, smallholder livelihoods, and food security across sub-Saharan Africa between 2016 and 2025.

Supplementary Table S1 shows a summary of the evidence on FAW exposure metrics, measured outcomes, and reported impacts on maize yield, household income, food availability, and nutrition in Africa and comparable maize-growing contexts. The table synthesizes findings from entomological surveys, field observations, household surveys, modelling studies, and literature reviews conducted mainly between 2016 and 2025. FAW exposure is captured using multiple proxies, including confirmed presence, infestation rates, larval counts, leaf damage scoring, farmer-reported infestation, and habitat suitability models. Yield loss percentages and infestation rates are reported where quantified; income, food availability, and nutrition effects indicate the direction of impact when assessed. Management practices reflect farmer responses or interventions discussed in each study, while limitations highlight key methodological constraints. These findings indicate that most studies primarily focused on agronomic outcomes, particularly yield loss and infestation rate, while livelihood and nutrition indicators were comparatively less frequently assessed.

### 3.6. Distribution of Reported Versus Inferred Outcome Data Across Empirical Fall Armyworm (FAW) Impact Studies

The distribution of reported and inferred outcome variables is presented in Table 4. Yield loss and infestation rate were the most consistently reported outcomes, reflecting the predominance of agronomic and entomological measurements in FAW research. In contrast, income and food availability were reported less frequently, while nutrition indicators were rarely measured. This pattern indicates a strong empirical evidence base for the biophysical impacts of FAW, alongside notable gaps in the direct assessment of livelihood and nutritional consequences.

### 3.7. Food Security Outcomes

Evidence from the reviewed studies indicates that *Spodoptera frugiperda* invasion has had substantial and persistent implications for food security in maize-dependent systems. Yield losses ranging from 20 to 50% directly translated into reduced household maize availability, particularly in Eastern and Southern Africa where maize constitutes the primary staple crop and alternative food sources are limited [5,14]. Higher FAW infestation rates, frequently exceeding 50%, were consistently associated with greater yield losses, indicating a positive relationship between infestation intensity and yield reduction. Although not quantified through meta-analysis, this pattern was consistently observed across empirical studies, supporting a clear directional link between pest pressure and productivity decline. Several household-level surveys reported declines in the number of months of maize self-sufficiency, increasing reliance on food purchases or external assistance during the lean season.

High infestation intensity, commonly exceeding 50% of fields, further exacerbated food insecurity by increasing the risk of partial or total crop failure, especially during the initial invasion period (2016–2017) when farmers had limited awareness and access to effective control options [1,2]. Rain-fed production systems were particularly vulnerable, as delayed or ineffective control during early crop growth stages resulted in irreversible yield losses.

Although food availability was frequently assessed, nutrition outcomes were rarely measured directly. Only four studies included indicators such as dietary diversity scores or food consumption measures, which generally declined during seasons of severe FAW infestation. These findings suggest that FAW impacts extend beyond caloric availability to affect diet quality, mainly through reduced income and diminished capacity to purchase diverse foods. However, the limited inclusion of nutrition indicators constrains a comprehensive assessment of FAW’s contribution to malnutrition and hidden hunger among smallholder households.

### 3.8. Livelihoods Outcomes

From a livelihood’s perspective, the synthesis of 20 studies consistently demonstrates that FAW infestation imposes significant economic burdens on smallholder farmers. Yield losses of 20–50% reduced both subsistence production and marketable surplus, directly lowering household income. Although precise quantification was not possible due to variability in reporting across studies, the available evidence suggests that yield losses of 20–50% translated into comparable reductions in marketable surplus, thereby directly lowering household income. In addition, FAW infestation increased production costs through expenditures on chemical pesticides and additional labor for scouting and control. The combined effect of reduced yields and increased input costs resulted in a substantial decline in net farm income, particularly among resource-constrained smallholder farmers. The most severe impacts were observed in regions with high maize dependency and limited access to affordable pest management options, reinforcing existing socioeconomic vulnerabilities [5,6].

Although a formal meta-analysis was not feasible due to heterogeneity in study design and outcome measurement, a consistent quantitative pattern emerges across the reviewed studies. High FAW infestation rates, frequently exceeding 50%, are generally associated with yield losses ranging from 20% to 50%, which in turn contribute to reduced household income. Approximately half of the included studies reported income effects, indicating a clear directional relationship between infestation intensity, yield loss, and livelihood impacts in smallholder maize systems.

Socioeconomic analyses revealed that FAW infestation increased production costs through heightened expenditure on chemical pesticides and additional labor for scouting and control. In many cases, these costs were incurred with uncertain effectiveness, further reducing net farm income. Farmers often responded by reallocating household labor from off-farm activities or other crops to FAW management, generating opportunity costs that were rarely quantified but likely substantial.

Multi-country studies highlighted that livelihood impacts varied according to infestation timing, climatic conditions, and farmer response capacity, including access to extension services and knowledge of integrated pest management strategies [6]. Households with limited financial and institutional support were less able to cope with repeated infestations, increasing their exposure to income shocks and long-term livelihood insecurity.

Overall, the evidence indicates that FAW invasion affects livelihoods not only through direct yield loss, but also through increased production costs, labor reallocation, and heightened economic risk. Nevertheless, the limited integration of agronomic, economic, and welfare indicators within individual studies restricts a full understanding of the cumulative and long-term livelihood consequences of FAW for smallholder farming systems.

These consistent yield impacts reinforce the importance of developing maize varieties with improved host-plant resistance, particularly in rain-fed smallholder systems where chemical control options are limited.

## 4. Discussion

The findings of this review confirm that FAW invasion has substantially affected maize production, food availability, and smallholder livelihoods across sub-Saharan Africa, consistent with earlier assessments [2,18]. The average yield reductions of 20–50% align with modeling projections for early infestation stages [15], suggesting that, despite increased awareness and control campaigns, the pest continues to threaten food security. While this review synthesizes evidence from studies conducted after the FAW invasion, broader maize production trends provide important context. Prior to the introduction of FAW in Africa around 2016, maize yields in many sub-Saharan African countries were relatively stable, although constrained by climatic variability and input limitations [1,2]. Following the spread of FAW, several reports have indicated increased yield variability and production risks associated with pest pressure [12]. However, direct comparisons between pre-invasion (e.g., 2015) and recent production levels (e.g., 2024/2025) remain limited and context-specific, as yield trends are also influenced by factors such as rainfall variability, input use, and policy interventions. This highlights the need for more longitudinal and nationally representative datasets to better quantify the long-term production impacts of FAW.

While the agronomic impacts are well documented, this review reveals significant gaps in livelihood and nutrition research. Less than half of the studies reported income or food availability outcomes, and fewer than 20% assessed nutrition-related indicators. This pattern mirrors the limited emphasis on socioeconomic evaluation in pest-management research, where most efforts prioritize agronomic performance and chemical efficacy [14,18].

The variation in yield loss and infestation rates across regions may be explained by differences in cropping systems, rainfall patterns, and management capacity. For instance, smallholders in Eastern and Southern Africa faced higher losses due to restricted access to pesticides, limited extension services, and delayed adoption of integrated pest management (IPM) approaches [5,6]. Conversely, West African regions that implemented early detection and biological control measures reported comparatively lower yield impacts [1].

A notable limitation across most studies is the absence of longitudinal designs, which prevents understanding of the long-term household recovery and resilience mechanisms following FAW invasion. In addition, heterogeneity in outcome measurement (e.g., different yield-loss estimation methods and inconsistent food security indicators) complicates meta-analysis and cross-country comparison.

To strengthen the evidence base, future research should integrate agronomic, socioeconomic, and nutritional perspectives, employing harmonized indicators and gender-disaggregated data. Policymakers should invest in community-based IPM programs, extension services, and regional monitoring systems that link pest surveillance to livelihood protection. Such approaches are essential for sustaining maize productivity and household food security in the face of persistent FAW pressure.

In addition, the synthesis of evidence presented in this review has important implications for maize genetic improvement programs. The widespread yield losses and persistent infestation levels reported across regions indicate that reliance on chemical control alone is unlikely to provide sustainable solutions. Host-plant resistance represents one of the most sustainable and cost-effective long-term strategies for managing fall armyworm in smallholder maize systems. Resistant varieties reduce reliance on chemical pesticides, lower production costs, and provide protection under resource-limited conditions typical of sub-Saharan Africa. Recent studies, including [12], have highlighted important advances in resistance mechanisms such as antibiosis, antixenosis, and tolerance, as well as progress in identifying resistant germplasm and developing improved maize hybrids. These developments are critical for enhancing the resilience of maize-based farming systems across Africa.

Despite these advances, the findings of this review indicate that empirical studies assessing the direct contribution of host-plant resistance to livelihood and food security outcomes remain limited. This highlights an important research gap and underscores the need for stronger integration between breeding programs, agronomic evaluation, and socioeconomic impact assessment in future FAW research. The geographic variability in reported impacts also suggests that breeding programs should prioritize locally adapted resistance traits, integrating phenotypic screening with molecular approaches to accelerate the development of resilient maize cultivars for African agroecologies.

## 5. Conclusions

This systematic review synthesized evidence from twenty-four empirical studies on the impact of FAW invasion on maize production, food security, and smallholder livelihoods in Africa. The findings demonstrate that FAW has caused substantial yield losses, increased production costs, and reduced household food availability across affected regions. While agronomic impacts are consistently documented, socioeconomic dimensions such as income reduction, coping strategies, and nutrition outcomes remain comparatively under-researched. Overall, the review highlights that FAW represents not only an agronomic challenge but also a broader food security and livelihood threat that requires integrated responses, including sustainable pest management, farmer support, policy coordination, and the development of resistant maize varieties as a long-term strategy for resilience in African smallholder systems.

## 6. Recommendations

1. Integrate socioeconomic and nutrition assessments into future FAW impact studies. Research should go beyond yield measurements to include income, dietary diversity, and household resilience indicators.
2. Standardize methodologies for estimating yield loss, infestation severity, and livelihood outcomes to enable cross-country comparison and meta-analysis.
3. Promote integrated pest management (IPM) approaches tailored to smallholder systems, emphasizing biological control, resistant maize varieties, and climate-smart practices [14,20].
4. Strengthen regional surveillance and early-warning systems to detect FAW outbreaks promptly and coordinate response actions.
5. Enhance farmer training and extension capacity through participatory programs that link pest control to improved household food security.
6. Encourage interdisciplinary collaboration among entomologists, agronomists, economists, and nutrition specialists to generate holistic evidence for policymaking.
7. Invest in policy frameworks that integrate pest management within national food security strategies, ensuring that interventions benefit the most vulnerable smallholder communities.

## Figures and Tables

**Table 1:** PICOTS framework used for study selection and evidence synthesis.

PICOTS Component	Description
Population	Smallholder maize farmers in Africa
Intervention	Exposure to fall armyworm infestation and associated management responses
Comparison	Pre- versus post-invasion conditions, affected versus less-affected areas, or different pest management strategies
Outcome	Impacts on maize yield, household food security, income, livelihood strategies, and coping mechanisms
Time	Studies published from 2016 to 2025
Setting	Smallholder and subsistence farming systems in sub-Saharan Africa

**Table 2:** Eligibility criteria used for inclusion and exclusion of studies based on the PICOTS framework.

PICO	Inclusion Criteria	Exclusion Criteria
Population	Smallholder maize farmers (household level) in sub-Saharan African countries affected by FAW	Studies conducted outside Africa (e.g., Latin America or Asia) even if they discuss FAW impacts on farmers
	Rural households or farming communities in African agro-ecological zones where FAW infestations have been reported (e.g., mixed cereal–maize systems)	Studies focusing only on large-scale commercial farms without data or analysis relevant to smallholder farmers
Intervention	Exposure to a documented FAW outbreak/invasion (e.g., sudden pest arrival, large seasonal infestation)	Studies focusing only on laboratory or screenhouse FAW biology (life cycle, genetics, host preference) without any link to food security or livelihoods
	FAW-related interventions or responses (e.g., emergency pesticide campaigns, introduction of biocontrol agents, farmer training/extension programs) implemented as a consequence of FAW presence	Research on pest management technologies (e.g., insecticide efficacy trials) that do not evaluate their impact on food security or smallholder livelihoods
Comparison	Before-vs-after comparison of the same community/households (pre-invasion vs. post-invasion)	Studies without a clear comparator (e.g., no before/after invasion data, or no affected vs. unaffected households/areas)
	Affected vs. unaffected areas or households (geographical or treatment/control comparison), or households using different management strategies (chemical control vs. integrated pest management)	Articles that compare FAW with other pests only, without isolating its impact on smallholder livelihoods or food security
Outcomes	Food security indicators—crop yield (kg/ha), household food availability, months of food sufficiency, dietary diversity score, or food expenditure	Studies that only report entomological outcomes (e.g., FAW population density, infestation rates) without linking them to household food security or livelihood measures
	Smallholder livelihoods indicators—farm income, proportion of crop lost to FAW (% loss), changes in labor allocation, asset sales, coping strategies, market access, or qualitative reports of livelihood disruption	Publications focusing solely on crop physiological responses (e.g., plant height reduction, leaf chlorophyll content) with no socioeconomic outcome measured

**Table 3:** Summary of included studies by geographic scope, countries covered, and type of research. The dataset comprises 20 studies, including country-specific empirical, multi-country empirical, and contextual studies (review, modeling, and grey literature).

Category	*n*	Countries/Region	Study Type
Country-specific empirical studies	16	Mozambique, Kenya, Zambia, Zimbabwe, Ghana, Uganda, Rwanda, Ethiopia, Malawi, Tanzania, Benin	Field studies, farmer surveys, household surveys, experimental and observational studies
Multi-country empirical studies	1	Ghana, Kenya, Uganda, Rwanda, Zambia, Zimbabwe	Multi-country survey/panel data analysis
Review/synthesis studies	1	Africa/sub-Saharan Africa	Literature review and synthesis (biology, ecology, impacts, and management)
Modeling/risk-mapping studies	1	Africa and beyond	Climate suitability and pest distribution modeling
Grey literature/program reports	1	Africa/sub-Saharan Africa	Institutional reports, program monitoring, and extension evidence
Total	20	—	—

**Table 4:** Summary of reported and inferred outcome variables across the 20 included studies.

Outcome Variable	Studies Reporting Data (*n*)	Studies with Inferred/Not Reported Data (*n*)	Reported (%)	Inferred/Not Reported (%)
Yield loss (%)	18	6	75	25
Infestation rate	17	7	71	29
Income	12	12	50	50
Food availability	11	13	46	54
Nutrition indicators	4	20	17	83

## Data Availability

All data supporting the findings of this systematic review are contained within the article and its Supplementary Materials. Additional extracted datasets, including study characteristics and geocoordinates used for geographic visualization, are available from the corresponding author upon reasonable request.

## Supplementary Materials

The following supporting information can be downloaded at https://www.mdpi.com/article/10.3390/insects17060589/s1, Table S1. Summary of included studies and supporting references used in the evidence synthesis [1,2,3,4,5,6,7,8,9,10,11,12,13,14,15,16,17,18,19,20].
