# Supplementary material for: Fall Armyworm in Maize: A Systematic Review of Smallholder Livelihood and Food Security Impacts in Africa
Source: Insects. 2026 Jun 4;17(6):589. doi: 10.3390/insects17060589 (PMC13300359; doi:10.3390/insects17060589)
Supplement: Supplementary file 1 [file insects-17-00589-s001.zip › insects-4198487-supplementary.pdf]

| Study | FAW exposure metric | Outcome(s) measured | Effect size / Key result | Management practices reported | Data collection year(s) | Yield loss % | Infestation rate | Income | Food availability | Nutrition indicators | Limitations |
|---|---|---|---|---|---|---|---|---|---|---|---|
| [1] | Confirmed presence / specimen identification | Occurrence, distribution (baseline) | First confirmed outbreaks in Africa; establishes invasion baseline | None (reporting identification) | 2016 | 20–50 | High (>50 %) | Not assessed | Reduced maize availability | Not reported | Descriptive; no household impact data |
| [2] | Literature synthesis, outbreak reports, surveillance data | Occurrence, spread, impacts, and implications for Africa | Foundational synthesis documenting the invasion, spread, and potential impacts of FAW across Africa; highlights major threats to maize production and food | Monitoring, pesticide use, IPM awareness, early response strategies | 2016 – 2017 | 20–50 | High (>50 %) | Reduced household income (projected/reported) | Reduced maize availability | Not systematically assessed | Contextual synthesis/report; not based on primary household survey or experimental data |

| | | | | | | | | | | | |
|---|---|---|---|---|---|---|---|---|---|---|---|
| | | | security | | | | | | | | |
| [3] | Climate suitability modeling, invasion risk projections | Potential distribution, invasion risk, and spread patterns | Predicted broad climatic suitability for FAW establishment across Africa and other regions; identified high-risk maize-growing areas vulnerable to invasion | Not primary focus; implications for surveillance and early warning systems | Model inputs from published and historical datasets | Not reported | Not reported | Not assessed | Potential implications for food security discussed | Not reported | Modeling study based on climatic assumptions; not based on field or household survey data |
| [4] | Field observations and specimen identification | Occurrence and confirmation of FAW infestation on maize | First confirmed report of FAW infestation on maize in India; documents pest establishment, distrib | Monitoring and pest identification emphasized | 2018 | Not reported | High (>50%) | Not assessed | Potential implications for maize production discussed | Not reported | Descriptive outbreak report; no household or food-security assessment |

| | | | | | | | | | | | |
|---|---|---|---|---|---|---|---|---|---|---|---|
| | | | ution, and invasion risk outside Africa | | | | | | | | |
| [5] | Infestation rates / field observations | Yield loss estimates | Empirical estimates of infestation and yield losses across Kenyan maize areas | Farmer practices and pesticide use reported | 2017-2019 (approx.) | 20–50 | High (>50 %) | Reduced household income | Reduced maize availability | Not reported | Context-specific; heterogeneity across sites |
| [6] | Damage scoring / infestation occurrence | Drivers of damage severity | Identifies factors explaining heterogeneity in damage | Cultural practices, scouting | 2017-2018 | 20–50 | High (>50 %) | Reduced household income | Reduced maize availability | Not reported | Observational; correlational |
| [7] | Farmer reports and field observation | Reported impacts, costs, coping strategies | Documents livelihood impacts and control costs | Pesticide use, cultural measures | 2018-2020 | 20–50 | High (>50 %) | Reduced household income | Reduced maize availability | Reduced dietary diversity | Localised study |
| [8] | Laboratory developmental observations under contro | Developmental duration, survival, and immature- | Provides standardized biological and developmental | Implications for pest monitoring and timing of control | Controlled laboratory study | Not reported | Not reported | Not assessed | Not assessed | Not reported | Laboratory-based biological study; no field, livelihood, or |

| | | | | | | | | | | | |
|---|---|---|---|---|---|---|---|---|---|---|---|
| | lled conditions | stage biological parameters | parameters of FAW immature stages, supporting understanding of pest ecology and life cycle dynamics | interventions | | | | | | | food-security assessment |
| [9] | Household survey (panel) | Yield, income, management | No significant effect of FAW on yield or income | Pesticides, biopesticides, IPM practices | 2018–2020 | Not significant | Moderate–high (declining) | No significant | Not assessed | Not assessed | Self-reported data; limited sample |
| [10] | Leaf damage scoring | Yield response to damage levels | Links leaf damage to yield loss; informs thresholds | Threshold-based recommendations | 2018-2021 | 20–50 | High (>50%) | Not assessed | Reduced maize availability | Not reported | Site-specific |
| [11] | Artificial infestation and leaf damage | FAW tolerance, damage severity, and varieta | Identified variation in FAW tolerance among | Host-plant resistance breeding and varietal | 2019–2021 (approx.) | 20–50 | Moderate to high | Not assessed | Reduced maize productivity | Not reported | Breeding-focused experimental study; limited direct |

| | | | | | | | | | | | |
|---|---|---|---|---|---|---|---|---|---|---|---|
| | scoring under field conditions | l response | maize parental lines, experimental hybrids, and commercial cultivars in Southern Africa; highlights potential sources of host-plant resistance | deployment | | | | | potential | | livelihood and food-security assessment |
| [12] | Infestation reports / field checks | Income, food security indicators | Quantifies FAW impacts on household income and food security metrics | Pesticide use, adaptation strategies | 2017-2019 | 20–50 | High (>50%) | Reduced household income | Reduced maize availability | Reduced dietary diversity | Self-reported measures |
| [13] | Literature synthesis / review evidence | Biology, ecology, impacts, and management strategies | Comprehensive synthesis of FAW impacts on crops, food | Integrated pest management, host-plant resistance, biologic | 2016–2025 | 20–50 | High (>50%) | Reduced household income (reported in synthesis) | Reduced maize availability | Not Systematically assessed | Review-based evidence; not primary empirical data |

| | | | | | | | | | | | |
|---|---|---|---|---|---|---|---|---|---|---|---|
| | | | systems, and management approaches in Africa | al control | | | | | | | |
| [14] | Not applicable | Reporting standards and methodological guidance for systematic reviews | Provides updated PRISMA 2020 guidelines for transparent reporting, study selection, screening, and synthesis in systematic reviews | Not applicable | Not applicable | Not reported | Not reported | Not assessed | Not assessed | Not reported | Methodological guideline; not an empirical or contextual FAW impact study |
| [15] | Literature synthesis and global evidence review | Crop impacts, yield losses, and action thresholds associated with FAW | Synthesizes global evidence on FAW impacts on crop productivity, estimated yield losses, and | Integrated pest management, monitoring thresholds, pesticide application, and cultural control strategies | Studies published up to 2020 | Variable; commonly 20–50% reported in affected regions | Moderate to high | Reduced agricultural productivity and potential income losses discussed | Potential implications for food security highlighted | Not systematically assessed | Review-based synthesis; not based on primary field or household data |

| | | | | | | | | | | | |
|---|---|---|---|---|---|---|---|---|---|---|---|
| | | | economic/action thresholds for management interventions | | | | | | | | |
| [16] | Not applicable | Quality appraisal and risk-of-bias assessment guidance | Provides standardized critical appraisal checklists for assessing methodological quality and risk of bias in included studies | Not applicable | Not applicable | Not reported | Not reported | Not assessed | Not assessed | Not reported | Methodological assessment tool; not an empirical or contextual FAW study |
| [17] | Not applicable | Risk-of-bias assessment framework for non-randomized studies | Provides the ROBINS-I tool for systematic assessment of bias in non-randomized intervention | Not applicable | Not applicable | Not reported | Not reported | Not assessed | Not assessed | Not reported | Methodological assessment tool; not an empirical or contextual FAW study |

| | | | | | | | | | | | | |
|---|---|---|---|---|---|---|---|---|---|---|---|---|
| | | | studies, supporting methodological rigor and evidence quality evaluation | | | | | | | | | |
| [18] | Household survey (multi-country) | Management, pesticide use, farmer response | High reliance on pesticides; unsafe use common | Pesticides, cultural, mechanical, IPM practices | 2018 | 12–58 (reported) | High (>50%) | Not assessed | Not assessed | Not assessed | Self-reported data; cross-country variability |
| [19] | Literature synthesis and regional evidence review | FAW invasion patterns, control practices, and resistance breeding strategies in sub-Saharan Africa | Summarizes FAW spread, farmer management responses, pesticide use, and progress in host-plant resistance breeding across sub-Sahara | Pesticides, cultural control, integrated pest management (IPM), and resistance breeding approaches | Studies published between 2016–2020 | Commonly reported range of 20–50% in affected areas | High (>50%) frequently reported | Reduced household productivity and economic impacts discussed | Reduced maize availability and production risks highlighted | Not systematically assessed | Review-based synthesis; limited primary empirical data and strong dependence on previously published studies |

| | | | | | | | | | | | |
|---|---|---|---|---|---|---|---|---|---|---|---|
| | | | n Africa | | | | | | | | |
| [20] | Program monitoring reports, surveillance summaries, and institutional response data | FAW spread, response coordination, extension support, and management interventions in Africa | Summarizes FAO-supported actions and regional responses to FAW invasion in Africa, including surveillance, farmer training, pest management promotion, and institutional coordination | Integrated pest management (IPM), farmer training, monitoring, pesticide stewardship, and extension support | 2017 – 2018 | Not systematically quantified | High (>50 %) reported in affected areas | Potential impacts on household livelihoods discussed | Risks to maize availability and food security highlighted | Not systematically assessed | Grey literature/program report; limited primary empirical data and peer-reviewed evidence |

Table S1. Summary of included studies

Reference


1. Goergen, G.; Kumar, P.L.; Sankung, S.B.; Togola, A.; Tamò, M. First Report of Outbreaks of the Fall Armyworm Spodoptera Frugiperda (J E Smith) (Lepidoptera, Noctuidae), a New Alien Invasive Pest in West and Central Africa. *PLoS One* **2016**, *11*, doi:10.1371/journal.pone.0165632.
2. Day, R.; Abrahams, P.; Bateman, M.; Beale, T.; Clottey, V.; Cock, M.; Colmenarez, Y.; Corniani, N.; Early, R.; Godwin, J.; et al. *Fall Armyworm: Impacts and Implications for Africa*; Research Information Ltd, 2017; Vol. 28;.

3. Early, R.; González-Moreno, P.; Murphy, S.T.; Day, R. Forecasting the Global Extent of Invasion of the Cereal Pest Spodoptera Frugiperda, the Fall Armyworm. *NeoBiota* **2018**, 25–50, doi:10.3897/neobiota.40.28165.
4. SHARANABASAPPA, C.M. KALLESHWARASWAMY, R.ASOKAN, H.M. MAHADEVA SWAMY, M. S. MARUTHI, H. B. PAVITHRA, KAVITA HEGDE, SHIVARAY NAVI., S.T. PRABHU, G.G. First Report of the Fall Armyworm, Spodoptera Frugiperda (J E Smith) (Lepidoptera: Noctuidae), an Alien Invasive Pest on Maize in India. *Pest Management in Horticultural Ecosystems* **2018**, *24*, 23–29.
5. De Groote, H.; Kimenju, S.C.; Munyua, B.; Palmas, S.; Kassie, M.; Bruce, A. Spread and Impact of Fall Armyworm (Spodoptera Frugiperda J.E. Smith) in Maize Production Areas of Kenya. *Agric. Ecosyst. Environ.* **2020**, *292*, doi:10.1016/j.agee.2019.106804.
6. Baudron, F.; Zaman-Allah, M.A.; Chaipa, I.; Chari, N.; Chinwada, P. Understanding the Factors Influencing Fall Armyworm (Spodoptera Frugiperda J.E. Smith) Damage in African Smallholder Maize Fields and Quantifying Its Impact on Yield. A Case Study in Eastern Zimbabwe. *Crop Protection* **2019**, *120*, 141–150, doi:10.1016/j.cropro.2019.01.028.
7. Makgoba, M.C.; Tshikhudo, P.P.; Nnzeru, L.R.; Makhado, R.A. Impact of Fall Armyworm (Spodoptera Frugiperda) (J.E. Smith) on Small-Scale Maize Farmers and Its Control Strategies in the Limpopo Province, South Africa. *Jamba: Journal of Disaster Risk Studies* **2021**, *13*, 1–9, doi:10.4102/JAMBA.V13I1.1016.
8. Montezano, D.G.; Specht, A.; Sosa-Gómez, D.R.; Roque-Specht, V.F.; Paula-Moraes, S.V. de; Peterson, J.A.; Hunt, T.E. Developmental Parameters of Spodoptera Frugiperda (Lepidoptera: Noctuidae) Immature Stages Under Controlled and Standardized Conditions. *Journal of Agricultural Science* **2019**, *11*, 76, doi:10.5539/jas.v11n8p76.
9. Tambo, J.A.; Kansiime, M.K.; Mugambi, I.; Agboyi, L.K.; Beseh, P.K.; Day, R. Economic Impacts and Management of Fall Armyworm (Spodoptera Frugiperda) in Smallholder Agriculture: A Panel Data Analysis for Ghana. *CABI Agriculture and Bioscience* **2023**, *4*, doi:10.1186/s43170-023-00181-3.
10. Chisonga, C.; Chipabika, G.; Sohati, P.H.; Harrison, R.D. Understanding the Impact of Fall Armyworm (Spodoptera Frugiperda J. E. Smith) Leaf Damage on Maize Yields. *PLoS One* **2023**, *18*, doi:10.1371/journal.pone.0279138.
11. Matova, P.M.; Kamutando, C.N.; Kutywayo, D.; Magorokosho, C.; Labuschagne, M. Fall Armyworm Tolerance of Maize Parental Lines, Experimental Hybrids, and Commercial Cultivars in Southern Africa. *Agronomy* **2022**, *12*, doi:10.3390/agronomy12061463.
12. Tambo, J.A.; Kansiime, M.K.; Rwomushana, I.; Mugambi, I.; Nunda, W.; Mloza Banda, C.; Nyamutukwa, S.; Makale, F.; Day, R. Impact of Fall Armyworm Invasion on Household Income and Food Security in Zimbabwe. *Food Energy Secur.* **2021**, *10*, 299–312, doi:10.1002/fes3.281.
13. Togola, A.; Beyene, Y.; Bocco, R.; Tepa-Yotto, G.; Gowda, M.; Too, A.; Boddupalli, P. Fall Armyworm (Spodoptera Frugiperda) in Africa: Insights into Biology, Ecology and Impact on Staple Crops, Food Systems and Management Approaches. *Frontiers in Agronomy* **2025**, *7*, 1–17, doi:10.3389/fagro.2025.1538198.
14. Page, M.J.; McKenzie, J.E.; Bossuyt, P.M.; Boutron, I.; Hoffmann, T.C.; Mulrow, C.D.; Shamseer, L.; Tetzlaff, J.M.; Akl, E.A.; Brennan, S.E.; et al. The PRISMA 2020 Statement: An Updated Guideline for Reporting Systematic Reviews. *Bmj* 2021, *372*.
15. Overton, K.; Maino, J.L.; Day, R.; Umina, P.A.; Bett, B.; Carnovale, D.; Ekesi, S.; Meagher, R.; Reynolds, O.L. Global Crop Impacts, Yield Losses and Action Thresholds for Fall Armyworm (Spodoptera Frugiperda): A Review. *Crop Protection* 2021, *145*.
16. (CASP), C.A.S.P. CASP Checklists, Oxford.

17. Sterne, J.A.; Hernán, M.A.; Reeves, B.C.; Savović, J.; Berkman, N.D.; Viswanathan, M.; Henry, D.; Altman, D.G.; Ansari, M.T.; Boutron, I.; et al. ROBINS-I: A Tool for Assessing Risk of Bias in Non-Randomised Studies of Interventions. *BMJ (Online)* **2016**, *355*, doi:10.1136/bmj.i4919.
18. Tambo, J.A.; Kansiime, M.K.; Mugambi, I.; Rwomushana, I.; Kenis, M.; Day, R.K.; Lamontagne-Godwin, J. Understanding Smallholders' Responses to Fall Armyworm (Spodoptera Frugiperda) Invasion: Evidence from Five African Countries. *Science of the Total Environment* **2020**, *740*, doi:10.1016/j.scitotenv.2020.140015.
19. Matova, P.M.; Kamutando, C.N.; Magorokosho, C.; Kutywayo, D.; Gutsa, F.; Labuschagne, M. Fall-Armyworm Invasion, Control Practices and Resistance Breeding in Sub-Saharan Africa. *Crop Sci.* **2020**, *60*, 2951–2970, doi:10.1002/csc2.20317.
20. FAO *Briefing Note on FAO Actions on Fall Armyworm in Africa*; 2018;
